# A qualitative study of bereaved family caregivers: feeling of security, facilitators and barriers for rural home care and death for persons with advanced cancer

**DOI:** 10.1186/s12904-020-00705-y

**Published:** 2021-01-08

**Authors:** Anne Sæle Barlund, Beate André, Kari Sand, Anne-Tove Brenne

**Affiliations:** 1 Department of Circulation and Medical Imaging, Faculty of Medicine and Health Sciences, Norwegian University of Science and Technology, Trondheim, Norway; 2 Cancer Department, Førde Central Hospital, Førde, Norway; 3 Department of Public Health and Nursing, Faculty of Medicine and Health Sciences, Norwegian University of Science and Technology, Trondheim, Norway; 4 Department of Health Research, SINTEF Digital, Trondheim, Norway; 5 European Palliative Care Research Centre, Department of Clinical and Molecular Medicine, Faculty of Medicine and Health Sciences, Norwegian University of Science and Technology, Trondheim, Norway; 6 Cancer Clinic, St. Olavs Hospital, Trondheim University Hospital, Trondheim, Norway

**Keywords:** Palliative care, Home care, Caregivers, Advanced cancer, Home death, End-of-life

## Abstract

**Background:**

For cancer patients and their family, an important factor that determines the choice to die at home is the caregivers’ feeling of security when caring for the patient at home. Support to caregivers from healthcare professionals is important for the feeling of security. In rural areas, long distances and variable infrastructure may influence on access to healthcare services. This study explored factors that determined the security of caregivers of patients with advanced cancer who cared for the patients at home at the end of life in the rural region of Sogn og Fjordane in Norway, and what factors that facilitated home death.

**Methods:**

A qualitative study using semi-structured in-depth interviews with bereaved with experience from caring for cancer patients at home at the end of life was performed. Meaning units were extracted from the transcribed interviews and divided into categories and subcategories using Kvale and Brinkmann’s qualitative method for analysis.

**Results:**

Ten bereaved caregivers from nine families where recruited. Five had lived together with the deceased. Three main categories of factors contributing to security emerged from the analysis: “Personal factors”, “Healthcare professionals” and “Organization” of healthcare. Healthcare professionals and the organization of healthcare services contributed most to the feeling of security.

**Conclusion:**

Good competence in palliative care among healthcare professionals caring for patients with advanced cancer at home and well- organized palliative care services with defined responsibilities provided security to caregivers caring for advanced cancer patients at home in Sogn og Fjordane.

**Supplementary Information:**

The online version contains supplementary material available at 10.1186/s12904-020-00705-y.

## Background

To be cared for, and to die at home is important to many cancer patients and their family caregivers (hereafter: caregiver) [1]. To be able to fulfil home death, support from caregivers is important [2]. They know the patient well and have the experience of how to help the individual. Many of the caregivers exert significant responsibility- and care tasks to their loved ones and perform a central part of the total care tasks given by the society [3–5].

However, to care for a cancer patient at home at the end of life is a considerable burden for the caregivers [6, 7]. Therefore, support to caregivers from healthcare professionals is equally important [8, 9]. Caregivers experience physical, psychological, social and financial challenges [2]. Many are not prepared for the demands and challenges they encounter [10]. The caregivers have to manage their own psychological worries, as well as the patients’ worries throughout the disease trajectory. At the same time, they have to provide help with daily tasks. Therefore, lack of focus on caregivers’ needs may lead to poor mental health and low quality of life [11]. The feeling of safety, stability, and being free from fear or anxiety through the disease trajectory gives security for caregivers [12]. Effective communication between healthcare professionals and caregivers empowers caregivers to fulfil their responsibilities and provide security to enable home care and home death [13]. This facilitates a smooth transition of care and allows the families to prepare for end- of- life phase and death [14].

Long distances, variable infrastructure and shortage of healthcare professionals- and services are barriers that make the access to specialized palliative care difficult in rural areas [15–18]. Palliative care is in many rural areas provided through standard nursing home services and general district nurses [19]. In Norway, palliative care for patients staying at home is provided by general practitioners and home care nurses. Palliative care beds are available in designated inpatient units in nursing homes in the municipalities [20–22]. District healthcare professionals therefore play an essential role in the provision of palliative care for cancer patients and caregivers with long distances to hospital and specialized palliative care services [19, 23].

Former Sogn og Fjordane county (merged with Hordaland county to Vestland county Jan 1, 2020) is a rural region in Western Norway. This is a region of more than 18,000 km^2^ with a small population of around 110,000. The region has two local hospitals and one central hospital. The region’s only palliative care team, as well as the oncology and haematology departments and the physicians responsible for cancer treatment, are located at Førde Central Hospital in the middle of the region. These consultants often use telephone contact or videocalls to conduct consultations with the patients, but when specialised treatments and examinations are necessary, the patients have to travel to Førde Central Hospital [24]. The longest drive from a municipality in Sogn og Fjordane to Førde Central Hospital is three and a half hours by car [25]. The region is characterized by fjords, mountains, and islands and has many narrow and winding roads with great danger of avalanches and mudslides, which often results in closed roads. Some municipalities are small, located on the mainland and have a short distance to the local hospital or have several nursing homes. Other municipalities are large, consist of several islands, and people may have to take a ferry or a boat to their destination, which also is dependable on the weather, or generally have a long travelling route. All this affects access to healthcare services.

The overall aim of this study was to explore factors that determined the feeling of security of caregivers of dying patients with advanced cancer that cared for the patient at home at the end of life in the region of Sogn og Fjordane in Norway.

The following research questions were addressed:

- What factors made the caregivers feel secure when they cared for the patient with advanced cancer at home in the rural region of Sogn og Fjordane?
- In the case that the patient died at home, − what facilitated home death?
- In the case that the patient did not die at home, − what were the barriers for home death?

## Method

### Study design

This is a qualitative retrospective study using semi-structured in-depth interviews with bereaved.

### Participants and recruitment

Participants were recruited between September and December 2018. Cancer coordinators and nurses working in ten municipalities and in the specialized palliative care team at Førde Central Hospital in Sogn og Fjordane received an email with written information about the study and a consent letter to present to potential participants. They contacted potential participants they had been involved with within the last 2–12 months. After the cancer coordinators/nurses had approached potential participants, the interviewer (ASB) contacted by telephone those who gave their permission to be contacted and gave them more information about the study. Interviews were conducted consecutively as the caregivers provided written consent to participate. More than one caregiver could be recruited per patient. In that case, they were interviewed together.

Inclusion criteria were parents, children or spouse 18 years or above of a deceased cancer patient; between 2 and 12 months since the loss of their loved ones; experience from being a caregiver of a cancer patient who did not permanently live in an institution at the end of life; experience with planned or unplanned home death or institutional death; previous or current contact with municipality cancer coordinator/nurse or palliative care team at Førde Central Hospital and ability to understand information in Norwegian or English. There were no exclusion criteria.

### Data collection and management

The interviews were conducted using a semi-structured interview guide developed by all of the researchers [26]. Three of the researchers (BA, KS, ATB) had experience from the participant population and from previous qualitative research [27–31]. The questions in the interview guide addressed informants’ experiences and thoughts from caring for the family member at home at the end of life, the experiences and interaction with healthcare services and what made them feel secure or not (Table 1). The interviews were audio recorded after consent from the informants, and transcribed verbatim. All the transcriptions were read and analysed independently by at least two of the authors [26]. Consensus regarding natural units, categories and subcategories during the analysis of the interviews was reached by discussion between the researchers. The demographic data about the informant and patient (Tables 2 and 3) were collected by a questionnaire filled in by the informants before start of the interview.

**Table 1:** Interview guide summary (Supplementary file 1)

• What was bothersome, rewarding, difficult by caring for the cancer patient at home?	
• Why did you choose to care for the patient at home?	
• What made you as a caregiver feel secure or insecure?	
• What was demanding?	
• What kind of expectation did you have?	
• How was the access to HCS and was this a problem?	
• Did you get support from others, and was this support crucial for caring for the patient at home?	
• What kind of support was most important?	
• Did you talk about death and preferences?	
• Was home death important for you and for the patient? Was the decision difficult?	
• How did you experience HCS?	
• What kind of offers and information did you get from HCS?	
• What was/ would have been the most important factors to take the patient home to die?

**Table 2:** Demographic characteristics of the informants *N*= 10

Age *mean (range)*	60,5 (41–76 years)
**Sex**	
Female *N (%)*	10 (100%)
Male *N (%)*	0
**Ethnicity**	
Norwegian	10 (100%)
**Highest level of education**	
Primary School *N (%)*	0
High School *N (%)*	5 (50%)
College/University *N (%)*	5 (50%)
**Living situation**	
Lived in the same residence as the deceased *N (%)*	5 (50%)
Lived in another residence than the deceased *N (%)*	5 (50%)
**Relationship to the deceased**	
Spouse/cohabitant *N (%)*	5 (50%)
Child *N (%)*	4 (40%)
Parent *N (%)*	1 (10%)

**Table 3:** Demographic and disease-related characteristics of the deceased *N*=9

Age *mean (range)*	72,9 (49–86 years)
**Sex**	
Female *N (%)*	2 (22%)
Male *N (%)*	7 (78%)
**Highest level of education**	
Primary School *N (%)*	1 (11%)
High School *N (%)*	5 (56%)
College/University *N (%)*	3 (33%)
**Years cancer-diagnosis** *mean (range)*	4,6 years (4 months- 18 years)
**Subcutaneous analgesia pump** *N (%)*	6 (67%)
**Months since death to interview** *mean (range)*	7,5 months (3–12 months)
**Place of death**	
Home *N (%)*	3 (33%)
Hospital *N (%)*	3 (33%)
Nursing Home *N (%)*	3 (33%)
**Contact with the palliative care team** *N (%)*	8 (89%)

### Analysis

The qualitative data analysis software NVivo 12 pro was used to import, manage and analyse the transcribed interviews. The analysis was performed according to the procedure described by Kvale and Brinkmann [26]. First, natural units were identified in the transcripts, and condensed into meaning units. Then codes were extracted from the meaning units. The codes were categorised inductively, i.e. the categories were derived from the data, not from the interview guide. Finally, subcategories were identified under each category, and illustrative quotes were selected.

### Ethics

The Regional Committee on Medical and Health Research Ethics (REK) determined that the study was in the category health services research and therefore did not need approval (2018/1129/ REK sør-øst). Health service research is not included in the Health Research Act and is therefore not obligated to get approval from REK [32]. The research protocol was approved by the Norwegian Centre for Research Data (NSD, ref.nr 61,366 AMS/LR) before start of the project.

## Results

Ten bereaved caregivers from nine families where recruited. They were all women, and they were all native Norwegians. Characteristics of the informants and the patients are given in Tables 2 and 3. Three main categories of factors were identified that made the caregivers feel secure when they cared for the patient with advanced cancer at home, or the opposite, lack of these factors made them feel insecure: Personal factors, healthcare professional factors and organizational factors (Table 4).

**Table 4:** Categories and subcategories identified to contribute to the feeling of security among caregivers of advanced cancer patients being cared for at home in former Sogn og Fjordane county

Categories	Personal factors	Healthcare professionals	Organization
Subcategories	Presence	Competence and knowledge	Healthcare service
	Attitudes towards the place of care and the place of death	Roles and responsibilities	Equipment
	To be more than one caregiver	Information	Norwegian Labour and Welfare Administration
	To talk about death	Language	Attendance allowance
	To have a driver’s license	Palliative care team, cancer coordinator and general practitioner (GP)	

### Personal factors

The informants talked about several personal factors that influenced the feeling of security when caring for the advanced cancer patient at home at the end of life. These factors varied from family to family and resulted in a different set of perceptions, attitudes and behaviour towards the feeling of security.

To be physically present and available for the patients in last phase of life was important to all the informants. One of the informants said it like this:

“*It was important to me to be with him... to be there, all the time. And it wasn’t hard, it was natural to be there. It was safe for me to be there with him” (Interview 6).*

The informants had different attitudes towards place of care and place of death. The most common reason given for home death, was that it was the patient’s wish to be at home. It was important for the caregivers to fulfil the last wish of the patient, even though it affected their own physical health, mental health or social life.

*“I knew he wanted me there, and that he felt safe because of this and calmed down. But really, I feel I went into vacuum. I lived on autopilot, I did what I was asked for, what was expected of me. I don’t know how I did it (...)I was unsure of a lot of things, and it was very stressful, certainly. But knowing that he felt safe, that was the most important thing to me” (Interview 5).*

At a hospital, it was said to be a restless pace, focus, and atmosphere. It was experienced as exhausting for both patient and caregiver. This made the informants feel stressful and they felt a lack of privacy and peace. At home, it was a calm atmosphere and more space to move in. This gave a greater feeling of freedom which contributed to the feeling of security.

“*( … ) because it is a completely different traffic in hospitals, and noise and- it’s tiring. ( … ) something had to be measured, something- blood tests or temperature or blood pressure or something … and if they were two-three patients in the room, then there was a lot of traffic” (Interview 6).*

However, if the patient’s situation was complicated with many issues and depending on many measures, some informants felt more secure when the patient was admitted to an institution with healthcare professionals (HCP) around all the time.

“*He wanted to be at home, but it didn’t work. He had so much tubing and drains and analgesic pump and epidural in his back and- ( … ) Yeah, I didn’t have the competence to manage that” (Interview 3).*

To have someone else to share the responsibility with of following the patient through the disease trajectory, discuss decisions concerning the patient and have the opportunity to share thoughts and worries, was said to be particularly important for the caregivers’ feeling of security:

“I declined the offer of attendance allowance; I wanted another sister to do this. I didn’t want to be alone in this [i.e. follow the patient in the last phase]. I wanted more people to be involved because I had- … *sigh* I had been doing this alone for so long” (Interview 5).

To be able to be open and realistic about the patients’ remaining time and that death was approaching was said to be a difficult topic, but important for the family concerned. By talking about this the informants could get to know the patient’s last wishes. Several of the informants wished that HCP brought up this topic:

“*I tried to hint, but I was so scared that it would be misunderstood, that he would think I would get rid of him or something, or that he would understand something else than what I really meant” (Interview 8).*

Informants settled around the county said that they were used to the long distances and had adapted to this. However, what was common for most of the informants and emphasized as an important factor for security was that they had a driver’s license:

“*It was of great help to have a driver’s license, because then I could come and go whenever I wanted to. Plus, then- I travelled often to our son because- it wasn’t always easy to come home to an empty house [i.e. when her husband was at the hospital]. You may have a need to be with others” (Interview 4).*

### Healthcare professionals

The need for qualified and helpful healthcare professionals (HCP) was most often said to contribute to the feeling of security. Qualified and helpful HCP comprise of the following topics: the need for knowledge and competence among HCP, clear distribution of roles and responsibility between caregivers and HCP, the need for information from HCP and the importance of speaking Norwegian. Specialised healthcare services like the palliative care team was appreciated, as was contact with the local cancer coordinator and the GP.

The informants felt safe when HCP had the knowledge to notice if the patient was in pain or in other ways unwell, and competence to provide medication or other measures to ease the situation. It was substantial for the caregivers that HCP not only approached the patient, but also turned to the caregivers, so they felt seen in this phase. Several of the informants expressed a need for talking to the HCP when HCP visited the patient because they had spent most of the day alone with the patient. They had therefore many questions and needed confirmation that what they were doing was right.

*“The home nurse didn’t ask, just went straight to the room. Didn’t ask how the night had been, very little dialogue. They didn’t care about my mother as a caregiver. She needed care. Someone to talk to. Someone to discuss medication with, that asked how the night had been and how the medication should be throughout the day, how much morphine we should administer” (Interview 1).*

The informants experienced that caring for the ill patient at home carried a lot of responsibilities. They felt like they had to take the responsibility for providing the right medication, get the necessary equipment, and make sure that the patient got the right care. Some expressed that they had to monitor the patient all the time because they were afraid that HCP would overlook something or not see changes when they were visiting, like changes in pain level or signs of infections. This led to less trust in HCP and a high level of stress, exhaustion, insecurity and less energy and concentration in the need of just having the role as a family member.

*“It felt safe and secure for us to know that they were visiting her [i.e. home nursing care]. But when changes occurred, they were slow. And in that phase of changing, it’s very tough to be a caregiver, because you see things, feel things and know things, and the rest [i.e. HCP] doesn’t follow” (Interview 2).*

Lack of information, unclear information, and information given too late was said to be a big problem that contributed to a great deal of insecurity among the informants. The informants said that since they never had experienced going through a palliative- and terminal phase before and were lacking knowledge about the patients’ course at the end of life, they did not know what to ask for or what to expect. They expressed that they did not get such information as what required hospital admissions like changing in pain level or signs of infections.

“*But it is like asking the right questions, knowing what to ask. What do you need to know? And you do not know this before you are in that situation, right? All the time it’s something new you want answers to” (Interview 8).*

Also, how the information was given was decisive. When the informants received brochures or other written information to read without HCP’s explaining, they often did not read it because they did not have time to read a brochure and/or felt that the information was not valid for them yet or that the information was given too late. This made the informants feel that HCP’s were unrealistic to where the patient was in the phase of the disease or that the informants themselves had denied accepting the outcome of the disease.

“The cancer coordinator came with these brochures … *laughter* about dying at home and everything and left them up there with my mother. I just took them away. Took them home with me, hid them. Like, “we shall not talk about this”” (Interview 5).

Some informants said that the information they got after treatment was not useful for them because they did not understand what it was saying:

“*The document he got after treatment: I wondered “what does this mean? What does it say?” Right? And they [i.e. HCP] may have explained a little bit, but you can't remember everything. It had been much better if they wrote things normal people understood, not these foreign words” (Interview 8).*

The lack of information about what to expect in the terminal phase was said to be of concern, especially when the patient was home and did not have daily contact with the specialist HCS. They said that information about what is normal in this last phase would have contributed to less concern and greater security. They also missed that the HCP were more open about death with the families.

“*All these changes, right? He was fine when he came home, ate and everything, and watching him from eating normally to doesn’t want to eat, doesn’t want to drink. It’s a process. When you haven’t been through it before, you don’t know what it is. Now I know that it is normal, this is how it is when a body starts to collapse: you stop eating, you stop drinking” (Interview 5).*

Some of the informants claimed that this made them make uninformed decisions:

“*I didn’t know what I went to. We didn’t know that we took him home to die, right? We knew very little about what we did ( … ) we didn’t know how much work it was going to be. The fact that it should always be someone with him, 24 hours a day, hadn’t thought about the consequences of that at all” (Interview 5).*

When the caregiver and HCP had an open communication and common understanding of the situation, this led to a feeling of security:

*“We had many conversations with the doctors and nurses at the hospital before we took him home [i.e. the patient] where we could ask any questions we wanted. I felt that we got very good information about the situation and of taking him home at the end of life” (Interview 9)*

To be able to understand what they were told by the HCP and to feel that they were being understood was important for the caregivers’ feeling of security. Several informants said that it was uncomfortable, and they felt insecure to be involved with HCP who did not master the Norwegian language:

“*( … ) if he said something, they answered “yes” no matter if they understood or not. So, both he [i.e. the patient] and I felt terribly insecure ( … ) No matter what you said, you never knew if they understood what you meant” (Interview 7).*

Some of the informants felt more secure when the palliative care team took over the treatment and care of the patient. However, they wished that the team came into the picture earlier in the palliative phase. Some of the patients had lived with incurable cancer for several years without contact with the palliative care team until the last months or days of life.

“*It was a shame that they were presented the last few days of mom’s life ( … ) They should have been present earlier ( … ) They don’t meet us [i.e. the family] at our best, the last few days” (Interview 2).*

### Organizational factors

Good organization of the palliative healthcare service (HCS) in Norway was important for caregivers to feel secure when carrying out palliative care at home. Subjects that were repeated in the interviews were interaction and collaboration between HCS in the municipalities and the hospital, the need for equipment and facilitation at home, support from The Norwegian Labour and Welfare Administration (NAV) and thus facilitation for use of attendance allowance. Many of the informants said that they could not have managed the caregiving without the service of home nursing care. However, it was considered as a burden and perceived as difficult to be involved with home nursing care when the caregivers experienced poor organization of the service:

*“They [i.e. home nursing care service] came with different competence: everything from unskilled persons to nurses. It seemed as if they had not considered what this patient needed of competence and continuity and what information they needed before they came into the home. Such an ill patient- I was very disappointed. But you must understand: the ones who came did their best, right? It wasn’t anything wrong with them, right? There was something wrong with the system” (Interview 1).*

It was also said that with home nursing care it was unpredictable when someone came and gave painkillers or provided other important measures:

*“( … ) you couldn’t ring a bell and get help when you needed to, it’s not the same with home nursing care, right? They are on their rounds and there are large geographical areas and they can’t get there so fast, so you feel much more alone. So, the insecurity with how we would get help when we needed it was- when could they come, right?” (Interview 1).*

The informants were dependent on access to competent healthcare services 24/7 to care for the terminally ill patient at home. When this was not possible, and when necessary measures could not be carried out at home, it created a barrier for home care. For example, home nursing care services in some municipalities could not administer intravenous interventions because of the lack of resources and/or competence. In these cases, admissions to nursing homes were necessary and contributed to caregivers’ feeling of security, even though it was not the patient’ or caregiver’s first choice in place of care.

*“When she [i.e. the patient] came home [i.e. from the hospital], she had to have intravenous pain relief, but the home nursing care couldn’t come to our home and administer this, so she had to go to the nursing home every day” (Interview 9).*

Good collaboration between the GP and home nursing care was said to be important for the implementation of home care and home death. Knowing that the family and home nursing care got help right away from the GP contributed to a feeling of great security, instead of having to contact the hospital or emergency room with unknown doctors who did not know the patient.

*“We always had the home nursing care in hindsight who was prepared to help us whenever we needed. It wasn’t a problem to call the GP either, if it was something, no matter what time of the day, but we didn’t call him, but we had the opportunity, and that was a safety for us to know” (Interview 6)*

A major concern the informants had in terms of having the patient at home was access to equipment. Cooperation between HCP at the hospital and in the municipality was said to be important in facilitating home care and get the necessary equipment in place.

*“They were incredibly good at the hospital [i.e. HCP]. We had an occupational therapist who submitted applications, facilitated the house and received the equipment we needed. They contacted the municipal physical- and occupational therapist who came home, and … We got what we needed.” (Interview 4)*

To be involved with NAV was repeatedly said to contribute to insecurity. NAV is the Norwegian social security system that manage unemployment benefit, pensions, attendance allowance and care allowance [33]. Informants experienced resistance and slow processing time from this authority, which led to greater insecurity in the care of the patient at home and the informant’s economic situation before and after the patient’s death:

*“They [NAV] said that they had to have a confirmation that my husband really was so sick ( … ) It wasn’t unfamiliar that he was sick, within the NAV– system, but this is the way it is. I haven’t heard from NAV since. They wait until they don’t have to pay. That’s what happens” (Interview 8).*

Although attendance allowance was said to be of good help when the informants had received information of this right, the system was said to be difficult and not adjusted to today’s cancer care or the caregiver’s everyday life.

*“( … ) but it’s a demanding system because you have to plan when to take out days and everything in that form, and you don’t know that, because it’s dependable on the health- both for my mother and my father [i.e. the caregiver and the patient]- so, therefore, it was barely used” (Interview 1).*

## Discussion

Personal factors among patient and caregivers and factors related to healthcare professionals and organization of healthcare were identified in this study to make caregivers feel more secure when caring for advanced cancer patients at home at the end of life in Sogn og Fjordane. The informants especially highlighted the importance of support from healthcare professionals and good organization of palliative healthcare services. Good communication between HCP and family/patient about patient matters and what to expect at the end of life, and between HCP so that HCS were well coordinated, was highly appreciated and contributed to security.

Contact between healthcare professionals, the patient, and the caregivers must be based upon understanding and trust [34]. To achieve this, it is essential that HCPs have knowledge and competence in the field, can provide timely and necessary information, and acknowledge and support the caregivers in their tasks. In Norway, every healthcare professional who work within the primary- or specialist healthcare is expected to have basic competence in palliative care [22]. Basic palliative care is part of HCP’s primary education [22]. In addition, nurses working with palliative patients should have additional competence in palliative care equivalent to level B. Level B is necessary competence to work with patients with palliative care needs and is provided through additional courses in palliative medicine and palliative care after primary education [22]. In rural districts, the recruitment of nurses with additional competence in palliative care may be difficult [35, 36]. This may result in palliative care services of lower quality in rural districts and increased likelihood of admission to hospital [16, 17]. It is also essential to have a clear distribution of roles and responsibility between HCP and the caregivers [8, 37–40]. Caregivers are often not used to care for severely ill patients and therefore have no previous knowledge about caring tasks, cancer or end-of-life care, which makes them dependent on HCP and HCS to fulfil the task [39, 41]. This is consistent with the findings in our study. Informants described that they felt safer when HCP with more experience or more education in palliative care or cancer care were involved; they could better answer questions and confirm that the caregivers did right.

Another finding in our study, is the importance of having Norwegian-speaking HCP in palliative care when the patients are native Norwegians. Our informants reported that poor language dissemination made them feel unsure about the HCP’s knowledge and competence. The barrier of language can prevent skilful nurses from being understood by their patients and colleagues. This can affect the patients’ and caregiver’s safety [42].

Earlier research and the present study revealed that the caregivers received information about the disease course when they requested it themselves [43]. Informants commented that they did not know what to ask about because they had not been through this before. This led to uninformed decisions, which is also reported by others [38, 43, 44]. Our informants expressed that they felt unsafe and unprepared for the role of caregiving when they were uninformed about what to expect or had an unrealistic expectation of care at home. It is shown by others that late information and the lack of information give the caregivers a feeling of uselessness, helplessness, insufficiency and a lack of control which again contributes to a great sense of insecurity [37–39, 41, 44, 45]. Our informants expressed that they were dependent on timely information about what they were getting into to be able to make rational and safe decisions.

To talk about the disease, prognosis, and death makes the caregivers more prepared for the end of life, the funeral and the time after death [46]. Still, many are afraid of hurting the patient by addressing these topics [44, 47]. Dealing with death and safeguarding the caregivers in the palliative phase and in the bereavement period are central elements in palliative care [48]. However, previous studies as well as this study found that these elements often are deficient in today’s organization of palliative care [44, 46]. Different caregivers have different needs and cope with information in different ways [44, 46]. Therefore, knowing the exact time and way to provide information to caregivers is not always clear. A valid evidence-based Carer Support Needs Assessment Tool (CSNAT) has been developed due to lack of practical tools to assess and evaluate caregivers’ support needs in the palliative phase [49]. By using this tool, individual caregivers’ needs for information and support can be improved and caregiving strains are reduced [49, 50].

According to Norwegian law, information about the patient’s health condition and the provided healthcare can only be given to the closest relatives, who often is the caregiver, if the patient gives consent [51]. Information distribution is therefore dependent on the patient. This safeguards the patient’s privacy. Still, according to Norwegian law, HCPs have a general duty of giving guidance to the caregivers [52]. Caregivers need information, advice, and guidance from HCP to be able to care for the patient at home [38], and this does not require consent from the patient. However, the caregivers are often in need of more than general information and guidance when they care for the patient at home, such as information about the patient’s prognosis or special considerations that must be taken. If the caregivers do not get this information, it can affect their possibility to prepare for death and thus make the experience of the palliative phase more difficult. For HCP, it may be an ethical dilemma that important information cannot be given to the caregiver due to confidentiality and privacy issues. The duty of guidance may therefore be neglected because HCP’s are afraid of breaking their duty of confidentiality. One way to overcome this, is to inform the patient and include the caregiver early in the disease trajectory [3]. We found in our study that if the caregiver and patient achieved a common understanding about the situation, the caregivers felt more secure and had fewer worries and unanswered questions. This supports involving the caregiver in addition to the patient when planning for the last phase of life.

Advanced Care Planning (ACP) is a communication process between the patient, the family and HCP where the patient plan for a time when he/she cannot make decisions for themselves. This includes reflection, deliberation and determination of the patient’s values, and wishes and preferences for treatment at the end of life [53]. None of our informants had experience with ACP. There is increased attention to the use of ACP in Norway [54], and this will maybe strengthen the involvement of caregivers.

Support from HCP and HCS helps mitigate the burden caregivers experience, which allows the patient to stay longer at home [13]. Continuity in home nursing care, close follow-up from the GP and contact with the Palliative care team at Førde Central Hospital were central elements in the organization of palliative healthcare that contributed to the feeling of security among the informants and facilitated home death. The informants reported that they felt security knowing that HCP came regularly. This is in line with results from another qualitative Norwegian study. This study, as ours, states that support from HCP is important when carrying out home care and home death for cancer patients [55]. It was important for the caregivers that HCP could perform necessary measures and that they could contact competent HCP whenever needed [55]. It also supports the need for available and predictable home nursing care to achieve continuity and thus security for the families who are caring for the patient at home.

Poor communication, interaction and organization between different healthcare professionals, healthcare services, the Norwegian Labour and Welfare administration (NAV) and families are barriers that can make it difficult to meet patients’ desires to stay home as long as possible and to die at home. Caregivers are not only dependent on HCP and HCS to fulfil home care and home death, but also other authorities like NAV. NAV helps with economic conditions for the patients who receive palliative care and their caregivers, as the arrangement of attendance allowance [22]. Attendance allowance allows a carer to care for the patient at home at the end of life. Knowing their economic rights give caregivers less concerns regarding their own working situation and makes it easier for the caregiver to be more present with the patient. The informants in our study experienced that NAV was difficult to reach and gave the caregivers unnecessary strain and insecurity, in line with a previous study [43]. Financial issues are often a barrier of completion of home palliative care [56, 57], because the caregiver has to leave work to manage the care at home [38]. Knowing about and receiving financial support, such as attendance allowance, may help to overcome this barrier.

Most of the informants in this study had access to a private car which contributed to a feeling of security caring for the patient at home, knowing they more easily had access to healthcare. Many people are dependent on owning a private car in Sogn og Fjordane because of the long distances and fewer offers of public transportation than in urban areas. Sogn og Fjordane thus lies in the upper half of the ranking of registered cars in Norwegian counties [58]. A study done in England in 2011 showed that distance was not a factor in the experience of hospital- accessibility [59]. The same study from England showed that the experience of access to HCS was significant related to socioeconomic status, and thus car-ownership [59].

Earlier studies have shown that the availability of a specialist palliative care team, both physically and for telephone contact, is important for the feeling of security at home [9]. The informants in this study reported that they missed that the team had been involved earlier. A study by Johnson et al. [60] showed that oncologists mainly refer patients with advanced cancer to specialized palliative care services for physical symptom-related reasons, and not for other reasons like psychological or social issues [60]. In addition, the oncologists stated that they were trained to take care of the physical symptoms of these patients, something that led to an even later referral to specialized palliative care services [60]. McDonald et al. (2017) found that caregivers of patients with advanced cancer who received early palliative care (6–24 months before death) were more satisfied with care than those receiving only standard oncological care [61]. These findings and the results in this present study support that early involvement from a specialist palliative care team and a more available team may contribute to better services for the caregivers as well as for the patients.

### Strengths and limitations

The qualitative research interview seeks to understand the world from the informant’s perspective and aims to produce knowledge within a topic [62]. Using a semi-structured approach, the interview provided room for the informants to deepen their views and provide additional information that they experienced as relevant to their situation.

Dependability and confirmability are major factors in understanding the implications of this study, and considerably effort was dedicated to examining these issues. Content analysis was used to identify similarities, differences, and patterns in the experiences of informants, and conclusions were deduced from the collected material without a predetermined hypothesis.

All the informants were from different municipalities with different distances to Førde Central Hospital.

All the interviews were analysed by minimum two researchers.

Many of the questions in the interview guide were formulated to address things that were positive in the palliative phase. During the interviews, the informants repeatedly drew attention to factors that were perceived as negative and contributed to additional insecurity instead of help in the palliative phase. This may have affected my analysis process. The opposite of factors that give insecurity are not necessarily factors that provide security and it could therefore be challenging to answer the research questions.

All the informants were women. One can only speculate on why there were no men included in the interviews. The interviewer was not involved in the actual recruitment of participants. Historically, women have had the main role of caregiving, and even today, women have easier to take on care tasks [63, 64]. One can therefore imagine that there was greater access to female informants during the recruitment process. It is uncertain how much the results were affected by the informants’ gender, but it cannot rule out that it had an impact. However, we could have controlled the recruitment process more by having more dialogue with cancer coordinators/nurses throughout the recruitment period about what kind of informants we wanted for this study.

## Conclusion and implications

This study from former Sogn og Fjordane county confirms the importance of support from healthcare professionals and a good organization of the palliative healthcare for giving the caregivers security in caring for the cancer patient at home in the last phase of life. It also confirms the importance of good coordination of care between the different services. Distance to hospital was not found to be a barrier for home care and home death. Many of the factors contributing to a feeling of security are factors that healthcare professionals can assess, map, and provide help with, in their interaction with the caregivers.

## Supplementary Information

**Additional file 1:.** Interview guide

## Data Availability

The audio-taped and transcribed interviews are not publicly available due to private details about the participants, but are available from the corresponding author on reasonable request.

## Abbreviations

REK: Regional committee on medical and health research ethics
NSD: Norwegian centre for research data
HCP: Healthcare professionals
HCS: Healthcare services
NAV: The Norwegian labour and welfare administration
CSNAT: Carer support needs assessment tool
ACP: Advanced care planning
