# Supplementary material for: A qualitative study of bereaved family caregivers: feeling of security, facilitators and barriers for rural home care and death for persons with advanced cancer
Source: BMC Palliat Care. 2021 Jan 8;20:7. doi: 10.1186/s12904-020-00705-y (PMC7796575; doi:10.1186/s12904-020-00705-y)
Supplement: Supplementary file 1 — Additional file 1:. Interview guide [file 12904_2020_705_MOESM1_ESM.docx]

# Interview guide

1. Can you tell me about what it was like for you to be a caregiver of an advanced cancer patient?

- What was the burden(s)?
- What was rewarding? What was important to you?
- What kind of feelings and worries did you have?
- What was most difficult in your role as a caregiver?

1. What was it that made you be there (as a caregiver) for ##, to the extent you did?
2. What contributed to the feeling of security in your role as a caregiver?

- Individual situations in phases/periods? What happened? What did you do? How did you experience it?

1. (Did you stay home with ##? (All the time? in periods?)) Was it at any time demanding to be home with ##?

- What was demanding?
- What / was it something that gave you a feeling of security in this situation?
- What / was it something that gave you a feeling of insecurity in this situation?
- What was demanded of you/what was your role in this situation?

1. Has it been different to be in the caregiver role than what you expected/envisioned? Did you have any expectations to this role, and if so - what?
2. Did ## spend time in a hospital/nursing home (in this palliative phase/when curative treatment was not offered)?

- In what situations was the patient in hospital? What was the reason the patient was admitted to the hospital?
- In what situations was the patient in a nursing home? What was the reason why the patient was admitted to the nursing home?

1. How was the access to healthcare services considering infrastructure, was this a problem? (ferries / boat, long journey)
2. Did you receive municipal services? (Home care nursing, cancer coordinator, etc.)
3. Did you or ## receive help/support from anyone other than healthcare professionals? (other family/friends/neighbors for e.g. shopping, transport, other tasks)

- Was help from anyone other than healthcare professionals decisive for ## staying longer at home?
- (Were you/## dependent on help from friends/neighbors for ## to stay at home?)

1. What help was most important when you were at home?

- Family, neighbours, friends, healthcare services, municipality, hospital
- Is there a long distance to other family, neighbours, friends, healthcare services, municipal centre, hospital?

1. How much did you (i.e. you and ##) talk in advance about where ## wanted to be at the end of life and/or where ## wanted to die?

- Did you know what ## wanted? Where did ## want to be at the end of life?
- Was it important for you to fulfil ## wish?
- Did you feel that ## wish pushed you more to fulfil this than you wanted yourself?
- Do you wish that you and ## had talked earlier about the end of life? (Where ## would be, planning, place of care, place of death)

1. Was it important to come home / be at home with ##? If so- what was important? (Important to come home to the home municipality / home to die?)
2. When ## died at home: Did you choose this/was this planned? Whose choice was it? Was this important for you? If so- why? How do you feel afterwards? Is there anything you wish was done differently? Do you have any positive experiences/thoughts about being at home? Do you have any negative experiences/thoughts about this? Was there anything that was difficult with this choice/in this situation? Did you feel more secure (for you/the patient) at home than in an institution? If so- why? What do you think had been different if ## was at an institution?
3. When ## died in an institution: Did you choose this/was this planned/do you feel that you had a choice? What was the reason for the choice/you did not have a choice? What were the barriers for ## coming home? Ev: Was it your decision, ## or did you together choose death in an institution? How do you feel afterwards? Is there anything you wish was done differently? What positive/negative experiences/thoughts do you have about this? Was there anything that was difficult with this choice/ this situation? Did you feel more secure (for you, for the patient) in an institution than at home? If so- why? What do you think had been different if ## was at home?
4. How have you experienced the meeting with the healthcare services?

- Do you feel there is good (enough) interaction/exchange of information between the hospital and the municipality in conjuction with discharge/admission? Have you felt confidence in that the municipality/hospital have been well (sufficiently) informed about needs and changes when ## was discharged/admitted?
- Have you had contact with the patient's GP? Has the GP participated in the planning of time at home/home death? Did the GP visit ## at home? If no: is this something you wanted?
- Have you had contact with your own GP (in this phase, about this/your needs)?
- Do you feel that you received (enough) information about the possibilities and arrangements for healthcare at home and possible home death? What kind of information did you get? By whom (municipality, palliative care team, doctor or similar)?
- Was there anything you dreaded in this role?
- Did you get information about who you could contact for help/questions?
- What information did you receive about your rights as a caregiver? For example about attendance allowance? Did you use this?
- What offer of follow-up did you (as a caregiver) receive from the healthcare services in ## last phase? What offer have you received in the time after ## death? If non- is this something you have missed? Is there anything else you have been missing?
- Were you offered to talk about worries/experiences? (Did the healthcare professionals contact you, and not just ##, when it came to thoughts about further planning/ expectations/ concerns/experiences?)
- Do you feel that you were well enough informed in advance about what awaited you in the role of being a caregiver at home?
- What is the most important support you have received?
- Is there anything you wish you got more information/support/help with?
- Is there something that was at no help at all/perceived as a burden?
- Has your experience changed your view of the Norwegian healthcare system? If yes- How and what?

1. Can you mention three key words that you think would have been the most important decisive factors (for you), if you were to take ## home at the end of life, that would give you a feeling of security? / Can you mention three key words that were the most important decisive factors (for you) to take ## home at the end of life, that gave you a feeling of security?

- Would you have recommended a friend (in the same municipality) to take their relative home at the end of life, if this friend were in a similar situation?

1. Is there anything else you want to add?
